# Editorial: Data-Enabled Intelligence for Medical Technology Innovation, Volume I

**DOI:** 10.3389/fmedt.2021.841150

**Published:** 2022-01-14

**Authors:** Nianyin Zeng, Kathy Clawson, Yonghong Peng

**Affiliations:** ^1^Department of Instrumental and Electrical Engineering, Xiamen University, Xiamen, China; ^2^School of Computer Science, University of Sunderland, Sunderland, United Kingdom; ^3^Department of Computing and Mathematics, Manchester Metropolitan University, Manchester, United Kingdom

**Keywords:** medical data analysis, digital health, data-enabled intelligence, artificial intelligence, machine learning, med-tech

Artificial intelligence (AI) is increasingly being applied to solve real-world problems across a variety of domains and provides opportunities to reshape healthcare, the economy, science, and beyond. In this context, AI- enabled Medical Technology (Med-Tech) has been gaining a significant amount of attention and interest. The development of Med-Tec that incorporate AI, big data methods, and Internet of Things (IoT) can enhance quality of life and has aroused academic interest within the global science community. There is increasing research focusing on how data-driven methods can be utilized to gain greater understanding of medical data, enhance decision making, and leverage operational efficiencies.

Med-tech is of significant importance in this big-data era—it facilitates the development of more advanced devices and corresponding algorithms in order to address global healthcare challenges. This Research Topic seeks original research articles in data science and artificial intelligence that extend medical technology innovation and facilitate a better understanding of the frontiers of AI methods in medical areas.

We are pleased to see the quality and volume of research that was submitted to our topic in data-enabled intelligence for medical technology. We received a total of 28 international submissions from scholars in a variety of countries (China, United States, Switzerland, Netherlands, Sweden, Italy, India, and Germany) and accepted 16 quality and most relevant articles. We provide a brief introduction of accepted papers herein, and welcome readers to refer to these papers and their associated references for more details on the topic. Liangming et al. proposed an obstructive sleep apnea patient rescue monitoring system which provided data support via a Beidou satellite system. Jin et al. presented a breast cancer-specific database and analysis of corresponding applications based on big data techniques. Li M. et al. employed a subtype and stage inference model to identify subtypes of knee osteoarthritis. Liang, Shi, et al. designed a Gaussian process model to predict knee joint angles via surface electromyography signals. Marini et al. developed a library of convolutional neural network (CNN) structures to extract multi-scale information from whole slide images.  Chen B.-q. et al. proposed a separation method on the basis of adaptive linear neuron, where the intrinsic structure of data was depicted by an autoregressive model. Liang, Ye, et al. designed a computer-aided detection (CAD) system based on faster R-CNN for early diagnosis of lung cancer via CT images. Chen B. et al. proposed an adaptive sparse detector for reducing power line interference based on sparse representation. Zhao et al. employed machine learning techniques to build a prediction model for large-scale screening of fatty liver disease. Lakkamraju et al. used fault-tolerant features in a non-invasive medical diagnostic framework to enhance system reliability. Anumukonda et al. designed an ANN-based multi-channel phonocardiography system for extracting cardiac sound components. Song et al. applied a two-step model and a human capital method to assess the economic burden of disease (EBD) in China. Shi et al. proposed a multi-input convolutional neural network to diagnose patellofemoral pain syndrome. Valle et al. developed a standard psychometric platform that could be used for data analysis in the somatosensory neuroprosthetics domain. Li Y. et al. carried out research on time irreversibility of HRV in a hypoxic environment. Wang et al. established an attention mechanism integrated long short-term memory (LSTM) model to predict Kellgren/Lawrence (KL) grade for knee osteoarthritis patients.

We would like to thank all authors again for their contributions to this special topic, and we very much appreciate the efforts of the reviewers for ensuring manuscript quality. It is highly hoped that this special topic could effectively advance the state-of-the-art innovations in medical technology.

## Author Contributions

NZ wrote the editorial. KC and YP edited the editorial. All authors contributed to the article and approved the submitted version.

## Funding

This work was supported in part by the Natural Science Foundation of China under Grant No. 62073271, International Science and Technology Cooperation Project of Fujian Province of China under Grant No. 2019I0003, in part by the UK-China Industry Academia Partnership Programme under Grant No. UK-CIAPP-276, in part by the Open Fund of Engineering Research Center of Big Data Application in Private Health Medicine of China under Grant No. KF2020002.

## Conflict of Interest

The authors declare that the research was conducted in the absence of any commercial or financial relationships that could be construed as a potential conflict of interest.

## Publisher's Note

All claims expressed in this article are solely those of the authors and do not necessarily represent those of their affiliated organizations, or those of the publisher, the editors and the reviewers. Any product that may be evaluated in this article, or claim that may be made by its manufacturer, is not guaranteed or endorsed by the publisher.

